# Classification of different degrees of adiposity in sedentary rats

**DOI:** 10.1590/1414-431X20155028

**Published:** 2016-02-23

**Authors:** A.S. Leopoldo, A.P. Lima-Leopoldo, A.F. Nascimento, R.A.M. Luvizotto, M.M. Sugizaki, D.H.S. Campos, D.C.T. da Silva, C.R. Padovani, A.C. Cicogna

**Affiliations:** 1 Departamento de Desportos, Centro de Educação Física e Esportes, Universidade Federal do Espírito Santo, Vitória, ES, Brasil; 2 Instituto de Ciências da Saúde, Universidade Federal do Mato Grosso, Sinop, MT, Brasil; 3 Departamento de Clínica Médica, Faculdade de Medicina, Universidade Estadual Paulista, Botucatu, SP, Brasil; 4 Departamento de Bioestatística, Instituto de Biociências, Universidade Estadual Paulista, Botucatu, SP, Brasil

**Keywords:** Obesity, Adiposity index, Rats, Cluster analysis

## Abstract

In experimental studies, several parameters, such as body weight, body mass index, adiposity index, and dual-energy X-ray absorptiometry, have commonly been used to demonstrate increased adiposity and investigate the mechanisms underlying obesity and sedentary lifestyles. However, these investigations have not classified the degree of adiposity nor defined adiposity categories for rats, such as normal, overweight, and obese. The aim of the study was to characterize the degree of adiposity in rats fed a high-fat diet using cluster analysis and to create adiposity intervals in an experimental model of obesity. Thirty-day-old male Wistar rats were fed a normal (n=41) or a high-fat (n=43) diet for 15 weeks. Obesity was defined based on the adiposity index; and the degree of adiposity was evaluated using cluster analysis. Cluster analysis allowed the rats to be classified into two groups (overweight and obese). The obese group displayed significantly higher total body fat and a higher adiposity index compared with those of the overweight group. No differences in systolic blood pressure or nonesterified fatty acid, glucose, total cholesterol, or triglyceride levels were observed between the obese and overweight groups. The adiposity index of the obese group was positively correlated with final body weight, total body fat, and leptin levels. Despite the classification of sedentary rats into overweight and obese groups, it was not possible to identify differences in the comorbidities between the two groups.

## Introduction

Obesity is characterized by excessive body fat accumulation. It is considered a global epidemic and constitutes a major public health concern (1). Although the etiology of obesity is complex, certain risk factors have been implicated in its development, including increased caloric intake and physical inactivity (2). Recent investigations have demonstrated that obesity decreases life expectancy and is associated with numerous medical complications, such as type 2 diabetes mellitus, dyslipidemia, and cardiovascular disease (3). To better understand the physiopathology of abnormalities associated with obesity, various animal models using either genetic or dietetic approaches have been proposed (4,5). Although genetic factors clearly contribute to the propensity of an individual to become obese, overconsumption of a high-energy diet may promote a positive energy balance and lead to overweight and obesity (6). Administering a high-fat diet to rodents reproduces many features of human obesity (7). Both humans and rodents tend to gain weight with high caloric intake (8). However, despite *ad libitum* feeding of a high-fat diet, obesity occurs in some but not all experiments (9,10).

Fat storage in humans can be easily estimated using various indicators, such as waist circumference, the waist/hip ratio, skin-fold thickness, and bioimpedance (1). However, body mass index (the ratio of weight to height^2^) is most often used to quantify body fat in humans (11). In experimental studies in rats, several parameters, such as body weight (BW) (12,13), body mass index (14), total body fat (15), fat pad mass (15,16), adiposity index (14,17), the Lee index (18,19), magnetic resonance imaging (20), and dual-energy X-ray absorptiometry (21), have commonly been used to demonstrate increased adiposity and investigate the mechanisms underlying obesity. Studies have observed energy intake, nutrient partitioning, and weight gain to describe processes that differ between rats that are either prone or resistant to becoming obese (22,23). However, these investigations have not experimentally classified the degree of adiposity because separating and identifying different adiposity intervals in animals is difficult, in contrast to classifying human obesity using the body mass index. Therefore, categories of adiposity in rats, such as normal, overweight, and obese, have not been defined in experimental studies.

Obtaining homogeneous groups of animals to measure their degree of adiposity is important, because it enables accurate diagnosis of experimental obesity and distinguishing between different parameters and comorbidities in these models. Because of the lack of studies that have classified the degrees of adiposity experimentally, the purpose of the present study was to characterize the level of adiposity in rats fed a high-fat diet (HFD) using the adiposity index. To achieve this objective, we used cluster analysis to identify the metabolic and nutritional profiles of the animals and create adiposity intervals in an experimental model of obesity. We hypothesized that a cluster analysis of data from rats fed a HFD for 15 weeks would allow us to distinguish various degrees of adiposity, similar to those defined in human obesity.

## Material and Methods

### Animal care

Thirty-day-old male Wistar rats were individually caged and subjected to different dietary regimens. All animals had free access to water and chow (50 g/day). After starting the experimental protocol, BW was recorded weekly. The environment was maintained under a 12-h light/dark cycle that started at 6:00 am, a clean-air room temperature of 23±3°C, and 60±5% relative humidity. All experiments and procedures were performed in accordance with the Guide for the Care and Use of Laboratory Animals published by the U.S. National Institutes of Health and approved by the Ethics Committee of the Faculdade de Medicina, Universidade Estadual Paulista, Botucatu, SP, Brazil (#565).

#### Diet and experimental protocol

The rats were distributed into two groups: normal diet (ND, n=41) and HFD (n=43). The ND group was fed a standard diet containing 12.3% kcal from fat, 57.9% kcal from carbohydrates, and 29.8% kcal from protein. The HFD group received a diet containing 49.2% kcal from fat, 28.9% kcal from carbohydrates, and 21.9% kcal from protein. The HFD was calorically dense (HFD=3.65 kcal/g *vs* ND=2.95 kcal/g) because of the increased fat content. The rats were maintained on their respective diets for 15 consecutive weeks and then euthanized. After 15 weeks of the dietary protocol, the data underwent cluster analysis for adiposity classification.

#### Experimental diet composition

The experimental diets provided sufficient amounts of protein, vitamins, and minerals according to the Nutrient Requirements of Laboratory Animals (24). The ND and HFD used in this study were formulated by Agroceres (Brazil). The ingredients were first ground and then mixed with vitamins and minerals. The mixture was formed into pellets, dried in a ventilated drying oven at 55±5°C, and stored at -20°C. The ND (RC Focus 1765) contained soybean oil, whole corn, wheat bran, soybean bran, dicalcium phosphate, sodium chloride, fish and meat flour, an antioxidant additive, and a vitamin and mineral mixture.

The HFD contained sodium chloride, casein, powdered milk, soybean protein concentrate, whole corn, cracker flour, dicalcium phosphate, calcium carbonate, emulsifier additives, antioxidants, flavoring, and a vitamin and mineral mixture. The HFD was formulated with a 0.9% concentration of sodium chloride and included saturated and unsaturated fatty acids that provided 20% and 80% of fat-derived calories, respectively. The high-unsaturated-fat diet consisted of 38% monounsaturated fatty acids and 42% polyunsaturated fatty acids, which were derived from the following fatty acids: 38% oleic acid, 41% linoleic acid (n-6), and 1% linolenic acid (n-3).

#### Adiposity index

The animals were anesthetized with sodium pentobarbital (50 mg/kg BW, *ip*), decapitated, and thoracotomized. The adipose tissue fat pads were then dissected and weighed. Total body fat was measured as the sum of the following individual fat pad weights: epididymal fat + retroperitoneal fat + visceral fat. The adiposity index was calculated as (total body fat/final BW) × 100 (25). The adiposity index was used as a measure of adiposity, because the degree of fat tends to increase gradually with obesity.

#### Cluster analysis of the degree of adiposity

Currently, there are no standard criteria for defining overweight or obesity in laboratory animals (e.g., rats and mice), companion animals, or other species. Using the adiposity index of animals fed a ND or HFD, we performed cluster analysis to establish similar groups with regard to adiposity level and to distinguish degrees of adiposity in these rats.

Cluster analysis is a statistical tool for classification and data reduction. This method allows large amounts of undivided data to be classified into subgroups based on similar characteristics (26). Methods such as k-nearest neighbor classification are useful exploratory tools for classifying relatively large amounts of data into subgroups when there is some prior knowledge of the potential number of subgroups within the given data set. For example, this method can be used to classify animals into either a normal-weight or obese group based on their phenotypic characteristics (e.g., BW) (27).

Using a set or unique parameter, the analysis generates a linkage tree that allows cases to be assigned to subsets, termed clusters. These clusters can then be grouped using the linkage method. A short linkage distance implies that the cases are similar. A long linkage distance implies that the cases are different. The method used in this study for data clustering was the closest neighbor technique (single linkage method), and the similarity coefficient used was the median Euclidean distance (26). The procedure used to analyze the conglomerates was the SAHN strategy for the formation of groups (SAHN involves “sequential, agglomerative, hierarchical, and non-overlapping” clusters). Finally, each cluster was described using dendrograms.

#### Determination of groups following cluster analysis

Following cluster analysis, the animals were allocated to groups according to their degree of adiposity. However, in biological experiments, particularly studies with animals, the results do not necessarily yield homogeneous groups (10). For this reason, the animals that received a HFD and exhibited changes in adiposity similar to the ND group were excluded from the analysis. The same procedure was performed with the rats that received a ND and exhibited changes in adiposity that were similar to the HFD group. These animals were rejected because of the necessity to characterize and distinguish adiposity from only obesity in rats that were fed a HFD and not in rats fed a balanced ND. After selecting the groups, the following characteristics were compared: nutritional state, systolic blood pressure (SBP), and metabolic and hormonal measures.

##### Nutritional analysis

Food consumption was measured daily, and BW was monitored once per week. Weekly caloric intake was calculated as the average weekly food consumption × the caloric value of each diet.

##### SBP

At the end of the experiment, SBP was assessed using the non-invasive tail-cuff method and a Narco BioSystems Electro-Sphygmomanometer (International Biomedical, USA). The average of two pressure readings was recorded for each animal.

##### Metabolic and hormonal measurements

At the end of the experimental period, the animals were subjected to 12-15 h of fasting, anesthetized using sodium pentobarbital (50 mg/kg BW, *ip*), and euthanized by decapitation. Blood samples were collected in dry tubes, and serum was separated by centrifugation at 3000 *g* for 15 min at 4°C and stored at -80°C for subsequent analysis. The serum was analyzed for glucose (GL), triglycerides (TG), total cholesterol (T-Chol), non-esterified fatty acids (NEFAs), insulin, and leptin. Serum GL, TG, and T-Chol concentrations were measured using an enzymatic automatic analyzer system (Technicon, RA-XT System; Global Medical Instrumentation, USA). NEFA levels were determined according to the method of Johnson and Peters (28) using colorimetric kits (WAKO NEFA-C; Wako Pure Chemical Industries, Japan). Leptin and insulin levels were determined using an enzyme-linked immunosorbent assay with specific commercial kits according to the manufacturer’s instructions (Linco Research, USA) (29).

#### Statistical analysis

Data from the cluster analysis, metabolic measurements, nutritional characteristics, and SBP values are reported as means±SD. These values are also reported as means and upper and lower limits of 95% confidence intervals for the mean. The results obtained from the cluster analysis were analyzed using one-way analysis of variance (ANOVA) followed by the Bonferroni *post hoc* test. Correlation analyses were conducted using the Pearson's linear correlation test. The level of significance was 5%.

### Results

**Figure 1:**
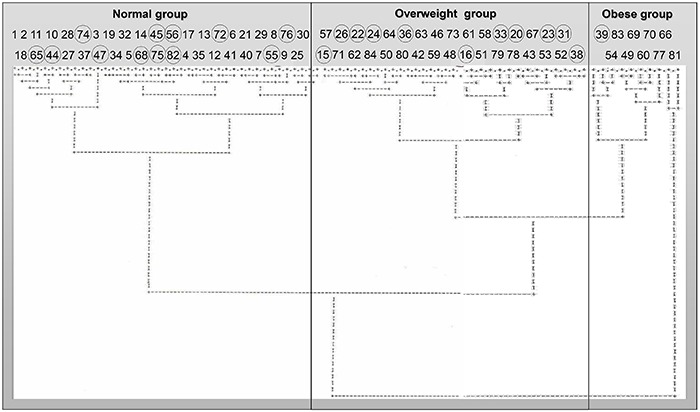
. Dendrogram of cluster analysis according to the adiposity index of control, overweight, and obese rats. Normal: animals from 1 to 41 that were fed a standard diet; overweight and obese: animals from 42 to 84 that received a high-fat diet. Animals represented by a circle were excluded from the study.

Following cluster analysis, 12 animals were excluded from each group because these rats displayed adiposity indices that were similar to the opposing group (Figure 1). Thus, the present study comprised 29 control animals and 31 overweight or obese animals. Furthermore, the cluster analysis allowed us to divide the rats that were fed a HFD into overweight (n=22) and obese (n=9) groups according to their degree of adiposity (Figure 1).

Tables 1 and 2 demonstrate that the overweight and obese groups exhibited significant differences in food consumption, final BW, visceral, retroperitoneal and epididymal fat pad mass, total body fat, and the adiposity index compared with those of the ND group. Furthermore, serum GL, leptin, and insulin levels were higher in the overweight and obese groups compared with those of the ND group. However, no differences in SBP or NEFA levels were observed between groups (Table 2). Additionally, caloric intake and T-Chol and TG levels were lower in the ND group compared only with those of the obese group (Tables 1 and 2).

**Table:**
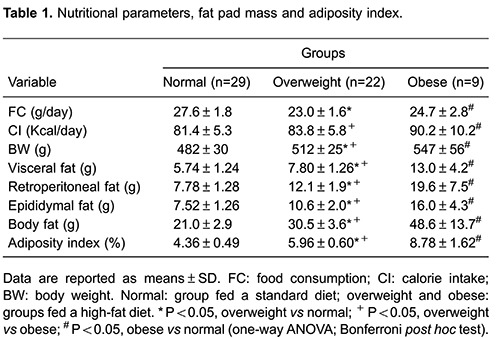

**Table:**
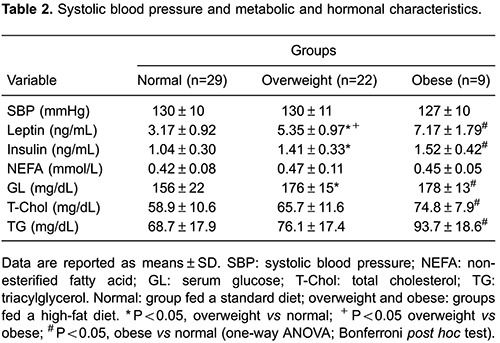

**Figure 2:**
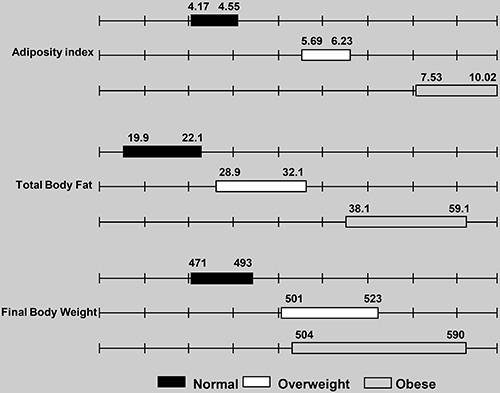
. Confidence intervals of adiposity index (%), body fat (g) and final body weight (g) for normal, overweight, and obese rats, grouped according to the cluster analysis technique. Normal: group fed a standard diet; overweight and obese: groups fed a high-fat diet.

**Figure 3:**
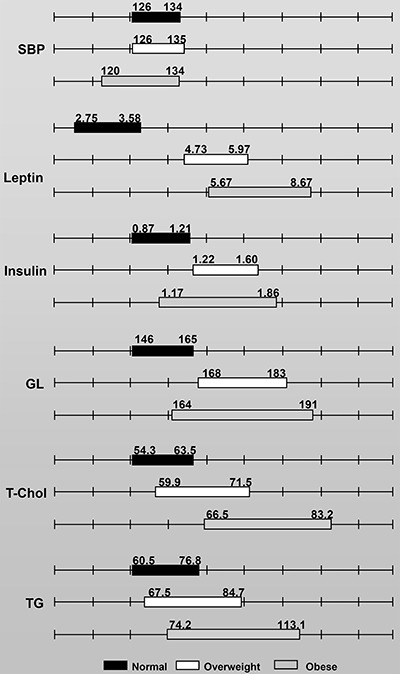
Confidence intervals of adiposity index and other variables for normal, overweight, and obese rats, grouped according to the cluster analysis technique. SBP: systolic blood pressure (mmHg); Leptin (ng/mL); Insulin (ng/mL); GL: glucose (mg/dL), T-Chol: total cholesterol (mg/dL); and TG: triacylglycerol (mg/dL). Normal: group fed a standard diet; overweight and obese: groups fed a high-fat diet.

Figures 2 and 3 report the confidence intervals for each group. After comparing the overweight and obese groups with the ND group, we identified no overlapping confidence intervals for the adiposity index, total body fat, or final BW (Figure 2). The confidence intervals for SBP and TG levels overlapped between the ND, overweight, and obese groups. However, no overlap was observed for leptin, insulin, or GL between the overweight and ND groups (Figure 3). The leptin or T-Chol level confidence intervals in the obese group did not overlap with those of the ND group.

Obese animals displayed a significantly higher final BW (P=0.03), total body fat (P<0.001), and adiposity index (P<0.001) than those of the overweight group (Tables 1 and 2). Additionally, fat pad weights were higher in the obese group than those in the overweight group (P<0.001). Although food consumption did not differ between the overweight and obese groups (P=0.076), caloric intake was greater in the obese group than that in the overweight group (P=0.04). No differences were identified in SBP or NEFA, glucose, T-Chol, or TG levels between the overweight and obese groups.

**Figure 4:**
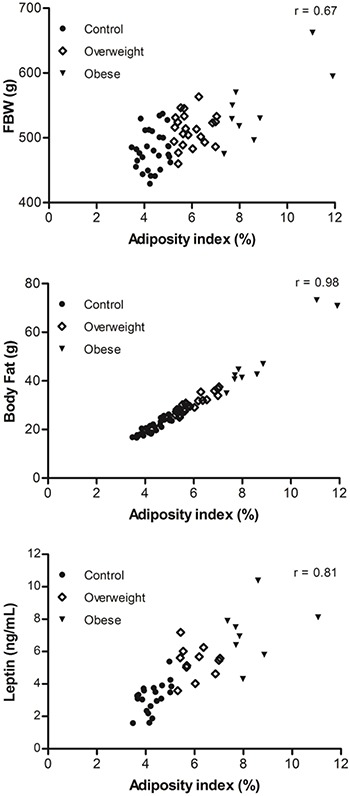
Correlations between adiposity index and final body weight (FBW), total body fat, and leptin levels after the 15-week experimental period.

The respective limits of the 95% confidence intervals are illustrated in Figures 2 and 3. No overlapping intervals for the adiposity index or total body fat were identified between the overweight and obese groups (Figure 2). However, overlapping confidence intervals for final BW, SBP, and leptin, insulin, GL, T-Chol, and TG levels were observed between the two groups (Figures 2 and 3). The adiposity index for all rats was positively correlated with final BW (r=0.67; P<0.05), body fat (r=0.98; P<0.05), and leptin levels (r=0.81; P<0.05) based on the Pearson's linear correlation test (Figure 4).

### Discussion

The diet used in the present study was of sufficient nutritional density and duration to promote obesity in rats. The contribution of caloric intake to obesity development in this model elicited significant differences in BW, body fat, fat pad mass, and the adiposity index between the obese and ND groups. The HFD also promoted many indicators that resemble those of human comorbidities caused by obesity, such as hyperinsulinemia, hyperleptinemia, and elevated serum GL, T-Chol, and TG levels. These results are consistent with several studies that induced obesity in rats by HFD-feeding (12,13,30).

The primary contribution of this study was the use of cluster analysis that allowed classification of degrees of adiposity in rats using the adiposity index, which is essential for studies that use experimental models to study obesity development. Cluster analysis is frequently used to stratify objects into groups according to similar characteristics (26,27,31,32). To our knowledge, this is the first study that has used cluster analysis to experimentally classify adiposity in an animal model of obesity. Furthermore, this technique allowed the creation of classification groups in accordance with the level of adiposity, similar to those defined for human obesity (i.e., overweight and obese) using the body mass index. This method enabled us to investigate the effects of varying degrees of obesity on nutritional, metabolic and hormonal parameters.

In studies using obese rats, the classification of obesity severity is limited. Researchers usually separate animals into those that are prone or resistant to obesity development (33 34 35 36), but they do not further sub-classify obesity-prone animals. In the present study, using the adiposity index, we demonstrated that obese rats exhibited different degrees of adiposity that could be classified into two groups. This heterogeneity is an important factor that may lead to discrepancies in experimental results because a relationship exists between the severity of obesity and its effects (10,15). Seeking one criterion that can be used to classify animals according to obesity severity, similar to the body mass index in humans, is necessary.

Cluster analysis did not demonstrate overlapping adiposity indices or total body fat between the overweight and obese groups. Body weight, an index that is often used as an indicator of obesity, exhibited overlap between the overweight and obese groups. However, the mean BW differed between the overweight and obese groups. Some previously published reports have demonstrated that BW can be a misleading indicator of obesity (10,15). In this case, BW greatly underestimated the actual degree of obesity that developed in obese rats. Our overlapping BW results confirmed that this variable alone is not a good indicator of adiposity.

Obesity is accompanied by many metabolic and hormonal changes, including glucose, T-Chol, TG, leptin, and insulin levels (12,13,30). However, in the present study the method used to classify overweight and obesity in rats was not effective at separating the metabolic and hormonal variables; insulin, leptin, GL, T-Chol, and TG confidence intervals overlapped between the two groups. Nevertheless, the mean leptin level was greater in the obese than in the overweight group. Previous studies have indicated that increased adiposity is directly associated with leptinemia (37,38). Considering that leptin promotes lipolysis, reducing TG uptake by adipocytes (38), hyperleptinemia probably increased serum TG levels in the obese rats. Importantly, hypertriacylglycerolemia is also associated with insulin resistance (38), which results in increased insulin levels to normalize blood glucose values, a condition called hyperinsulinemia.

Another parameter that is commonly impaired in obesity is arterial hypertension (19), although in this study alterations in SBP were not present. SBP confidence intervals also overlapped between the overweight and obese groups. Previous studies have reported an association between salt intake and increased blood pressure in experimental animals fed high levels of salt (19,39,40). Dobrian et al. (19) demonstrated that both 2% and 4% NaCl in a HFD induced a rapid increase in SBP in obesity-prone but not obesity-resistant or control rats. The absence of arterial hypertension in the overweight and obese groups in the current study may have been because of the low NaCl concentration (0.9%) in the HFD. Because the confidence intervals of many variables (metabolic, hormonal, and cardiovascular) overlapped between the two groups, we can confirm that these parameters were not accurate as individual markers of the degree of obesity in rats. Therefore, the joint or isolated use of variables that do not overlap will allow the classification of obesity in rats into two different degrees of adiposity.

As described in the results section above, some variables overlapped between the normal, overweight, and obese groups, although the mean values were significantly different. Such overlap can occur because of the variability in animals, which results in non-homogenous groups. Thus, animals that were fed a ND exhibited variable responses, and some animals even displayed characteristics that resemble those of obese animals. Although overlapping confidence intervals occurred among the control, overweight, and obese groups, the results indicated that for the extreme points, the normal and obese group means differed for almost all variables, most likely because of the high degree of adiposity in the obese group. Only SBP and NEFA levels did not differ between the three groups. Our study also confirmed that the adiposity index and other markers of obesity were positively correlated (Figure 4). These results suggest that the adiposity index is a good indicator of obesity, similar to several other studies that have used the adiposity index as an indicator of fat accumulation (14,19,36).

In conclusion, cluster analysis of the data obtained from sedentary rats subjected to 15 weeks of a HFD allowed the rats to be divided into two different categories of adiposity (overweight and obese). However, identifying differences in the cardiac and metabolic parameters between these two groups was not possible. Further studies with more prolonged periods of obesity are necessary to evaluate the different degrees of obesity and their potential association with disorders that resemble human comorbidities.
